# Magnetoelectric behavior via a spin state transition

**DOI:** 10.1038/s41467-019-11967-3

**Published:** 2019-09-06

**Authors:** Shalinee Chikara, Jie Gu, X.-G. Zhang, Hai-Ping Cheng, Nathan Smythe, John Singleton, Brian Scott, Elizabeth Krenkel, Jim Eckert, Vivien S. Zapf

**Affiliations:** 1 National High Magnetic Field Lab (NHMFL), Los Alamos National Lab (LANL), Los Alamos, NM 87545 USA; 2 University of Florida, Gainesville, FL 32611 USA; 3 Chemistry Division IIAC, LANL, Los Alamos, NM 87545 USA; 4 Material Science and Technology MST-11, Los Alamos, NM 87545 USA; 5 Harvey Mudd College, Claremont, CA 91711 USA

**Keywords:** Solid-state chemistry, Ferroelectrics and multiferroics

## Abstract

In magnetoelectric materials, magnetic and dielectric/ferroelectric properties couple to each other. This coupling could enable lower power consumption and new functionalities in devices such as sensors, memories and transducers, since voltages instead of electric currents are sensing and controlling the magnetic state. We explore a different approach to magnetoelectric coupling in which we use the magnetic spin state instead of the more traditional ferro or antiferromagnetic order to couple to electric properties. In our molecular compound, magnetic field induces a spin crossover from the S = 1 to the S = 2 state of Mn^3+^, which in turn generates molecular distortions and electric dipoles. These dipoles couple to the magnetic easy axis, and form different polar, antipolar and paraelectric phases vs magnetic field and temperature. Spin crossover compounds are a large class of materials where the spin state can modify the structure, and here we demonstrate that this is a route to magnetoelectric coupling.

## Abstract (web summary)

Magnetoelectric effect enables versatile electronic and spintronic devices. Here the authors demonstrate a different strategy to achieve magnetoelectric coupling in which the electric polarization is controlled by magnetic spin state transition instead of the traditional ferro- or antiferromagnetic order.

## Introduction

Magnetoelectrics are materials where the electric polarization can be sensed or controlled by a magnetic field and/or the magnetic properties by an electric field^1–4^. The interplay of spin, charge, and lattice needed to create such magnetoelectric coupling is an intriguing challenge and a source of creative discoveries. Magnetoelectric coupling is attractive for technological applications such as low power, tunable frequency devices, magnetic sensors, energy harvesting, computing, and data storage^5–7^. In most magnetoelectric and multiferroic materials, the magnetism consists of ordered patterns of the spin direction such as ferromagnetism, spiral antiferromagnetism, or more disordered behaviors such as paramagnetism or spin glasses^2,8,9^. Spin-state transitions (SSTs), also known as spin crossovers, on the other hand, provide a different magnetic functionality that has not been extensively experimentally investigated in the context of magnetoelectric or multiferroic-type behavior^10,11^. At a SST, the order parameter is the size of the total spin, not its direction. Magnetic field-induced SSTs can be co-operative (long range), switchable and hysteretic, and have enormous effects on the lattice up to and including triggering structural phase transitions. Therefore, SSTs open up a different route to achieving similar functionalities as magnetoelectrics and multiferroics.

SSTs can occur in transition metal ions with 3*d*^4^–3*d*^7^ electronic configurations. Electrons move between *d* orbitals^12,13^ to change the total number of unpaired electrons and thus the total spin.

SSTs are an extremely active area of research for magnetic materials containing organic ligands, where they tend to occur between room temperature and 10 s of Kelvin^12–21^. On the other hand, SSTs rarely occur in inorganic oxides at or below room temperatures and pressures^22–26^. Thus magnetic materials with organic ligands are the primary materials in which SSTs can be found in large numbers at temperatures below and up to room temperature and ambient pressures. SSTs manifest macroscopically as a significant jump in the magnetization. For example, an **S** = 1 to **S** = 2 transition can be expected to roughly double the magnetization. Since the *d*-orbital occupation is modified, the effective shape and size of the magnetic ion locally changes by up to 10%. Thus SSTs can produce substantial changes in the crystal structure, lattice parameters, dielectric, optical, and mechanical properties of the material and are often sharp and hysteretic. SSTs can be induced and influenced by external parameters including temperature, magnetic field, light irradiation, pressure, and chemical adsorption^14–20,27^. In this work we explore whether an SST can also toggle electric polarization and thereby create magnetoelectric coupling.

**Fig. 1:**
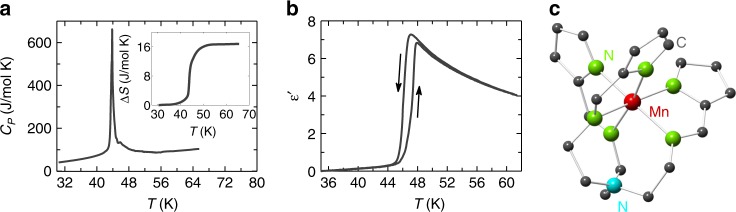
Physical properties of Mn(taa) at the temperature- induced SST. **a** There is a sharp peak in the heat capacity *C*_*p*_ vs temperature *T* at the SST in Mn(taa). The inset shows the corresponding entropy change Δ*S*. **b** The dielectric constant $\epsilon \prime$ as a function of temperature also shows a sharp transition at the SST above which Curie-Weiss-like paraelectric behavior continues up to room temperature in our data and the literature^26,31^. **c** A molecule of Mn(taa) with Mn^3+^ in a quasi-octahedral Nitrogen (green) environment. A seventh non-bonded Nitrogen is shown in blue. The standard errors due to random noise are smaller than the line widths

We investigate the insulating molecule-based magnet [Mn^3+^ (pyrol)_3_(tren)], also known as Mn(taa)^28^. This was one of the first Mn^3+^ based materials to show an SST. With increasing temperature *T* or magnetic field *H* the spin transitions from the low spin (LS) **S** = 1 ($t_{2g}^4$, $e_g^0$) to the high spin (HS) **S** = 2 ($t_{2g}^3$, $e_g^1$) state as an electron changes orbital^29,30^. The *T*-induced SST at 48 K is illustrated in the heat capacity *C*_*p*_(*T*) and the dielectric constant $\epsilon \prime (T)$ data in Fig. 1a, b, which we remeasure here for our crystals. The hysteretic, first-order nature of the SST in this compound implies that the spin states are coupled to each other. The lack of exchange coupling or ferroic order down to 4 K^31^ implies that the coupling mechanism is mainly lattice deformation^14,27,32^ as is common in molecule-based SST materials. The *T*-induced SST produces a ~1% change in the overall lattice parameter^33^ in Mn(taa) while preserving the average space group.

Mn(taa) crystallizes in a non-centrosymmetric, nonpolar $I\bar 43d$ cubic crystal structure with sixteen molecules per 20.3 Å unit cell^28,33,34^. Figure 1c shows one molecule of Mn(taa) where the Mn^3+^ ion occupies a trigonally distorted octahedron of nitrogen ions within a molecule. Supplementary Figure 1 shows the full crystal structure. The trigonal axes of the molecules point along the four different body diagonals of the cubic unit cell, creating four sublattices of four molecules each. Each molecule carries an electric dipole along its trigonal axis, and in the LS state these static electric dipoles cancel out when summed over all the molecules in the four sublattices. However in the HS state, dynamic Jahn-Teller (JT) distortions^29,34,35^ deform the molecules such that they contain an additional electric dipole component that is perpendicular to the trigonal axis. This distortion can occur in three different nearly degenerate ways, creating three directions for the HS dipole. These JT distortions and their associated dipoles fluctuate, creating the paraelectric behavior that appears in the HS (**S** = 2) state as shown in Fig. 1b. A Curie-Weiss fit to the dielectric constant yields a Curie-Weiss constant on the order of tens of Kelvin, implying ferroelectric interactions between the electric dipoles in the HS state^29,34^. The dynamic nature of these HS electric dipoles ensures that the time-averaged space group detected by X-ray crystallography remains the same in the HS and the LS state with no net electric polarization. The scenario described above has emerged from measurements of X-ray diffraction, magnetization, heat capacity, electron spin resonance, optical absorption, inelastic neutron diffraction, dielectric properties, and Raman scattering together with density functional theory^29–31,33–41^.

In dynamic JT systems, the fluctuating JT distortions often freeze in as *T* is lowered, triggering a phase transition to a new space group. This process is known as a cooperative JT effect^42^. If this were to occur in Mn(taa), the electric dipoles could possibly freeze into a net polar configuration creating a large electric polarization. In Mn(taa) however, such a cooperative JT effect is interrupted by the LS state, which intervenes and removes the fluctuating JT distortions before they can freeze in. The associated loss of electric dipoles creates a sharp drop in the dielectric constant at the SST, which we’ve remeasured for our crystals in Fig. 1b. On the other hand, if we apply a magnetic field at low temperatures, we could stabilize the HS state and its associated electric dipoles even at low temperatures, thereby allowing such an electrically polar state. The SST in Mn(taa) can be induced at *T* < 48 K by magnetic fields ranging from 10T up to at least 60T, depending on the temperature and the magnetic field sweep rate^37,40,41^.

Here we investigate the ordering of electric dipoles created in the HS state and their coupling to the magnetic field. Note that Mn(taa) belongs to a piezoelectric space group and the additional contribution from this effect to the electric polarization is also discussed below. We map the electric and magnetic properties of Mn(taa) with temperature and magnetic field and develop a theoretical model.

## Results

### Magnets and magnetic properties

**Fig. 2:**
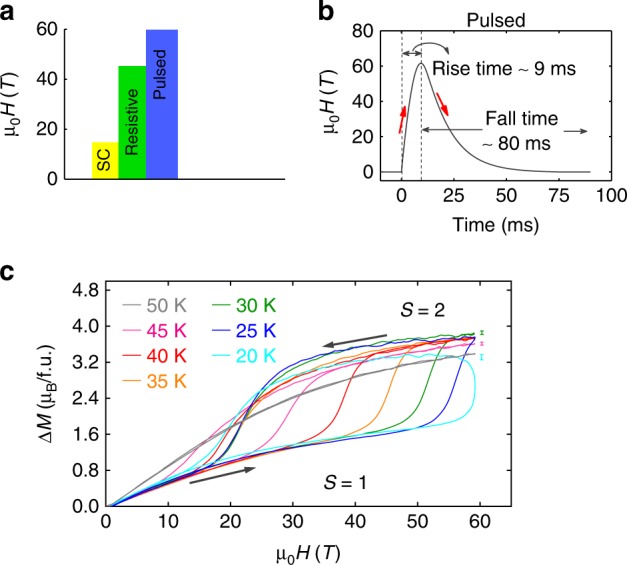
SST in Mn(taa) as a function of pulsed magnetic field. **a** Magnetic field range of the magnets used in this work: superconducting (up to 14T), resistive DC (up to 45T), and pulsed (up to 60T). **b** The magnetic field vs time for the pulsed magnet. **c** Δ*M* relative to *M* (*H* = 0) as a function of *H* up to 60 T for different *T* on a randomly oriented collection of single crystals. Data is taken after zero field cooling (electric and magnetic fields) from room temperature. The SST is evident as a broad jump in the magnetization. For a given temperature, the jump in SST occurs at a higher *H* for the upsweep than the downsweep. The standard errors due to random noise are smaller than the line widths. A systemic error due to mechanical vibrations yields oscillations, and their maximum amplitude is indicated by an error bars in the plot

Measurements were performed in low and high magnetic fields using quasi-DC and pulsed magnets. To extract equilibrium experimental properties we used quasi-DC magnets up to 14T to measure magnetization, dielectric constant and heat capacity, and quasi-DC magnets up to 45T to measure the change in electric polarization, Δ*P* (*H*, *T*) and dielectric constants $\Delta \epsilon \prime (H,T)$. The quasi DC magnets were either static or had a sweep rate between 0.08 and 0.17 Ts^−1^. We also performed measurements in millisecond pulsed-field magnets up to 60T with sweep rates up to 6 kTs^−1^. As pulsed fields reach higher magnetic fields and higher speeds compared to quasi-DC fields, they enable sensitive magnetization measurements Δ*M*(*H*) at high fields, and enhance the signal to noise ratio in electric polarization measurements^43,44^. However, the speed of pulsed fields can shift the fields at which the first order SST is observed and thus DC field data is important to extract the equilibrium phase diagram^37,40,45^. Thus we combine three types of magnets to reveal the full picture: quasi-DC superconducting magnets up to 14T, quasi-DC resistive and resistive/superconducting hybrid magnets up to 45T and millisecond pulsed magnets up to 60T, summarized in Fig. 2a, b.

In Fig. 2c we plot the magnetization Δ*M*(*H*) up to 60T in pulsed magnetic fields, showing evidence of the SST. With increasing *H*, *M* approaches the 2 *μ*_*B*_ saturation magnetization of the **S** = 1 state, then jumps to a Brillouin-like behavior in the **S** = 2 state above the *H*-induced SST approaching 4 *μ*_B_. This data is taken on a collection of randomly oriented single crystals. The hysteresis in the SST is about 30T between up and down sweeps of the pulsed field at 30 K. As *T* is lowered, the SST is pushed to higher *H* and can no longer be observed below 15 K in a 60T magnetic field pulse, consistent with the previous pulsed-field measurements^37,40^.

### Electrical properties

**Fig. 3:**
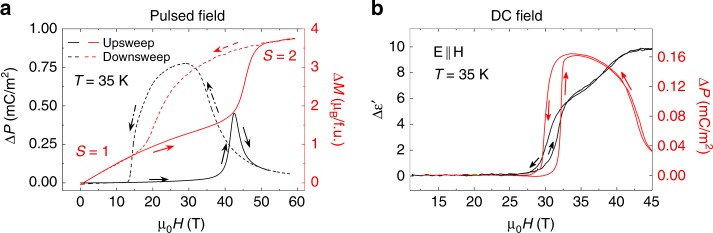
The change in electric polarization, magnetization, and dielectric constant at the SST. **a** Pulsed-field electric polarization Δ*P*(*H*) (left axis, black) and change in magnetization Δ*M*(*H*) (right axis, red) at *T* = 35 K for increasing and decreasing pulsed magnetic fields. Here **E** || **H** || [**110**] where **E** and **H** are the direction of the electric polarization and magnetic field. The magnetization is measured on a randomly oriented collection of single crystals. **b** Quasi-DC field dielectric constant $\Delta \epsilon \prime (H)$ (left axis, black) and electric polarization Δ*P*(*H*) (right axis, red) for increasing and decreasing quasi-DC fields. The loss tangent for dielectric measurements is less than 10^−5^ and **E** || **H** || [**110**]. The standard errors due to random noise are smaller than the line widths except for DC dielectric constant data, where the data are averaged, and the standard error is indicated

**Fig. 4:**
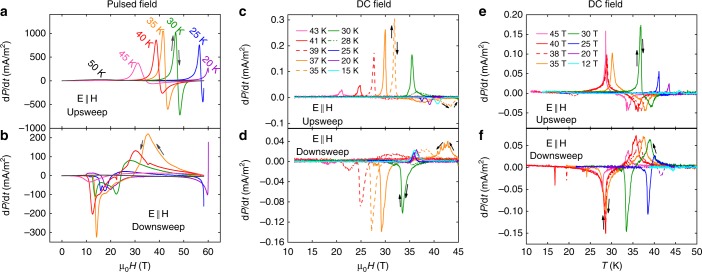
Magnetic field-induced electric polarization change d*P*/d*t* for **E** || [110] in pulsed and quasi-DC magnetic fields. **a**, **b** d*P*(*H*)/d*t* for different *T* during the upsweep and downsweep of the pulsed magnetic field *H*. **c**, **d** d*P*/d*t* measured in quasi-DC magnetic fields up to 45T at different *T* for the upsweep and downsweep. **e**, **f** d*P*/d*t* as a function of temperature in fixed magnetic fields up to 45T for **E** || **H **|| [**110**]. We see similar results for **E** ⊥ **H**, indicating no linear magnetoelectric coupling. The magnetic field and temperature of the peak in d*P*/d*t* is used to generate the data points in the phase diagram of Fig. 5. The error in estimating this peak due to random noise in this data is smaller the size of the data points in Fig. 5

**Fig. 5:**
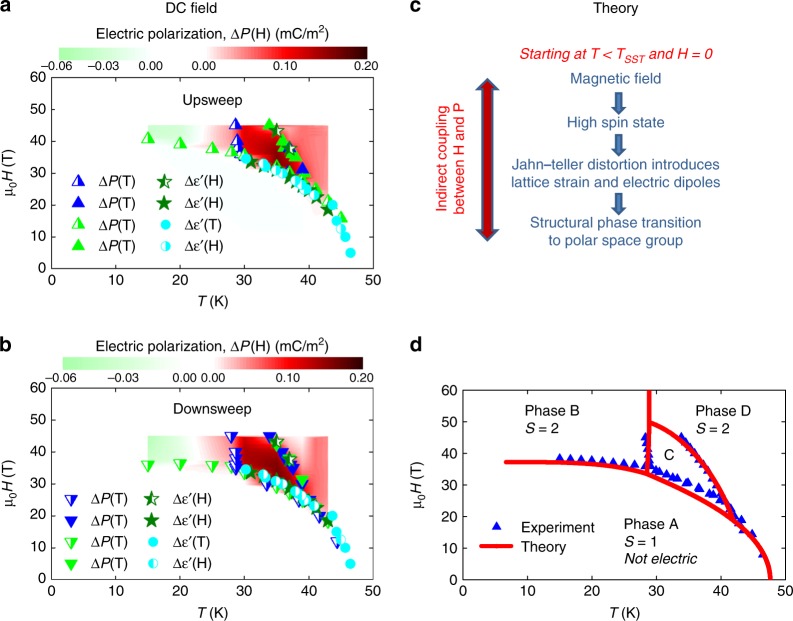
**a**, **b** Magnetic field *H* vs temperature *T* phase diagram of Mn(taa) for **E** || **H** in quasi-DC magnetic fields for up- and downsweeps. The color-mapped regions show the surface charge change relative to the low spin state. In the green region, Δ*P* switches sign relative to the red region. The data points in **a**, **b** are phase transitions defined as the inflection points in Δ*P*(*H*, *T*) and $\Delta \epsilon \prime (H,T)$. **c** Outline of the magnetoelectric coupling process for creating a net electric polarization in the high spin state. **d** Calculated mean-field phase diagram compared to experimental data points averaged between up and downsweeps of the field. The error bars are calculated by averaging over multiple measurements and samples and are smaller than the data points

In Figs. 3–5 we describe the electrical measurements Δ*P*(*H*,*T*) and $\Delta \epsilon \prime \left( {H,T} \right)$, with additional data shown in Supplementary Figs. 2–5, demonstrating the existence of strong magnetoelectric coupling in Mn(taa).

Figure 3a compares Δ*P*(*H*) and Δ*M*(*H*) in pulsed fields at *T* = 35 K. These quantities are obtained by integrating the measured d*P*/d*t* and d*M*/d*t* signals with respect to time. This figure shows that the onset of Δ*P* occurs at the the same magnetic field as the onset of the SST in the magnetization. *P* significantly decreases again at the highest magnetic fields where the spins saturate. Figure 3b compares Δ*P*(*H*) and $\Delta \epsilon \prime (H)$ in quasi-DC fields also at *T* = 35 K. Once again Δ*P* becomes finite at the SST in applied *H*, and then drops to a lower value at the highest *H*. In Supplementary Figs. 2 and 3 we show additional Δ*P*(*H*) and $\Delta \epsilon \prime (H,T)$ for different *T* and different orientations of the electric polarization (**E**) and the magnetic field **H**. We find that the shape of $\Delta \epsilon \prime (H,T)$ at the phase transition shows a dependence on the orientation of **E** with respect to **H**, but shows no change in the transition field or temperature.

In Fig. 4 comprehensive electric polarization data are shown. Fig. 4(a)–(d) show d*P*/d*t* measured at fixed *T* while sweeping *H* and Fig. 4 (e) and (f) show d*P*/d*t* at fixed *H* while sweeping *T*. Pulsed and DC field data are shown as labeled. The measurements are made with the configuration **E** || **H** || **[110]**. We find similar results for **E** || **[110]** ⊥ **H** shown in Supplementary Figs. 2–4, with identical transition fields and temperatures, and slightly different shapes of *P*(*T*, *H*). d*P*/d*t* is several orders of magnitude larger in pulsed fields than DC fields since it is proportional to the magnetic field sweep rate, while *P* differs only by a factor of 3 as discussed later.

The physical properties at the SST show significant hysteresis between increasing and decreasing *H*, consistent with a first-order phase transition. In the quasi-DC measurements in Fig. 4(c)–(f), the hysteresis in the transition field between *H*_transition_(up) − *H*_transition_(down) has a value between 1 and 3T depending on temperature, whereas in pulsed fields the hysteresis width can range up to 30 T. We found that the magnitude of the hysteresis in quasi-DC fields has no dependence on d*H*/d*t* between 0.017 and 0.08 Ts^−1^. These hysteresis trends are consistent with the first order displacive structural phase transition that would be necessary to transition between a nonpolar and a polar structure. First order phase transitions typically have slow dynamics since the nucleation and growth process must compete with the speed of the magnetic field, and thus have greater hysteresis in kTs^−1^ pulsed magnetic fields compared to sub-Ts^−1^ quasi-DC fields.

### Phase diagram

Figure 5(a), (b) summarize the phase diagram determined from quasi-DC electric polarization and dielectric constant measurements for the **E** || **H** data. Data for **E** ⊥ **H** yield an identical phase diagram as shown in Supplementary Fig. 4. The pulsed-field phase diagram is shown in Supplementary Fig. 5. In Fig. 5a, b, the color map shows Δ*P* (*H*, *T*) data obtained by integrating d*P*/d*t* in time. The overlaid data points are extracted from inflection points in Δ*P* (*H*,*T*) and $\Delta \epsilon \prime (H,T)$. Figure 5c summarizes a proposed mechanism for magnetic field-induced electric polarization due to the frozen JT electric dipoles discussed in greater detail below. Figure 5d compares the phase diagram calculated by this mechanism to the experimental phase diagrams. The different magnetic and electric phases are labeled A (LS), B (HS at low *T*), C (HS at intermediate *T*), and D (HS at high *T*).

As can be seen in Fig. 5(a), (b), the SST is strongly first order and hysteretic, and follows a roughly mean field-like shape in *H* − *T* space. At the lowest temperatures, *T* < 15 K in phase B, the signatures of the *H*-induced SST in all the measurements fade away in quasi-DC measurements, and are pushed above 60T in pulsed data, shown in Supplementary Fig. 5. The fact that the SST is pushed to higher fields at higher magnetic field sweep rates is typical for a first order phase transition where the speed of nucleation and growth competes with the speed of the changing magnetic field. Effectively we see a supercooling effect, except as a function of magnetic field instead of temperature. This effect is also seen in other field-induced SST materials^45^. As *T* is increased above 15K (phase C), d*P*(*H*)/d*t* changes sign and increases by a factor of ten at a vertical phase boundary at *T* = 28 K. This boundary is observed both in *T* and *H* sweeps and shows minimal hysteresis. As the temperature is further increased, a slanted phase boundary occurs between 35 and 40 K that also shows no resolvable hysteresis. Above this temperature in phase D, the static electric polarization decreases significantly and the dielectric constant (response of electric polarization to electric fields) increases markedly. The dielectric constant vs temperature shows paraelectric Curie-Weiss behavior, suggesting that the static electric dipoles of phase C become unfrozen in phase D. Thus we show that in the *H*-induced HS state, Mn(taa) exhibits at least three phases (B, C, and D) with different electric properties for different regions of *T* and *H* separated by non-hysteretic phase boundaries. On the other hand, the SST itself between the LS (phase A) and the HS state is strongly hysteretic and first order.

## Discussion

There are two possible mechanisms for creating electric polarization in Mn(taa). One is due to the piezoelectric effect since the *H* = 0 structure of Mn(taa) belongs to a cubic piezoelectric space group. The second possibility is the freezing of JT electric dipoles in the HS state. To investigate the piezoelectric contribution, we measure the SST as a function of temperature at *H* = 0 and observe an electric polarization change of 0.02 mC m^−2^ (Supplementary Fig. 6). Since the crystal structure at *H* = 0 is known to be cubic and nonpolar both above and below the *T*-induced SST, this electric polarization can be attributed entirely to the piezoelectric effect. The SST induces a 1% change in lattice parameters, thus it is likely that the crystal experiences strain due to the mismatch between the crystal and its substrate that induces the piezoelectric effect. A comparable electric polarization is also seen in the *H*-induced SST from phase A to phase B suggesting a similar mechanism. However in phase C, the electric polarization is 10 times larger and of opposite sign from phase B. We note that phase C occurs just as the electric dipoles that were paraelectric in phase D are now freezing in, evidenced by a sharp drop in the dielectric constant. Phase C is bounded by 2nd order-like non-hysteretic phase transitions to phase B and phase D. At these phase transitions, the dielectric constant shows a peak typical of ferroelectric type ordering (Supplementary Fig. 2). Thus we suggest that in phase C, the HS electric dipoles order into a polar configuration. This evidence supports the idea that in phase C the SST has induced a structural phase transition into a polar space group in which the JT electric dipoles are partially ferroelectrically aligned. The crystal structure does not allow a full ferroelectric alignment due to the different molecules in the four sublattices of the unit cell having different orientations. Thus a more complex ordering must take place.

Artifact exclusion due to conductivity and other effects is discussed in the S.I. on these tera-ohm insulating samples. We note that our data is reproducible when the measurements are repeated for a given sample and the overall phase diagram is reproducible between five different samples as shown in Supplementary Fig. 4. However, we find irreproducibility in the peak magnitude of the electric polarization in phase C. The peak electric polarization value varies between measurements on different samples from 0.35–1.2 mC m^−2^. We attribute this variation to the fact that the material must spontaneously choose a polar axis from among the equivalent cubic axes. Since we can’t predict this choice, the polar axis in phase C will vary with respect to our measurement axis. Significantly, we find that the largest observed Δ*P*(*H*) of 1.2 mC/m^2^ is within a factor of ten of the largest Δ*P*(*H*) for any organic or inorganic system^46–48^.

In Fig. 5c we summarize our proposed mechanism for the electric polarization in phase C wherein paraelectric electric dipoles in the HS state freeze in to form a state with net electric polarization. We have modeled this process using a mean field theory and plot the calculated phase diagram in comparison with the experimental data in Fig. 5d. The experimental data points are averages between the up and down sweeps in Fig. 5a, b. Our theory, detailed in the Supplementary Note 2 and Supplementary Discussion, is based on a 4-state Potts model and is an extension of the theory by Nakano et. al. for Mn(taa)^34^ and Kimura et. al. for Mn(taa)^40^. The four states *ρ*_*i*_, *i* = 0, 1, 2, 3 are the **S** = 1 state (*i* = 0), and the three different ferrodistortive **S** = 2 states that have electric dipoles pointing in different directions relative to their molecules. Our extension of this model also accounts for the four sublattices of molecules that point in different directions, and finally our model includes free energy terms in the electric polarization. The four phases A–D in Fig. 5d are: A. LS phase: *ρ*_0_ = 1, *ρ*_1_ = *ρ*_2_ = *ρ*_3_ = 0. B. HS phase with net *P* = 0: *ρ*_0_ = 0 and one of *ρ*_1,2,3_ ≈ 1, with a **P** = 0 due to ordering on different sublattices. C. Polar HS phase *ρ*_0_ = 0, *ρ*_1_ = *ρ*_2_ = *ρ*_3_ = 1/3, distributed across the four sublattices so as to create net **P** ≠ 0. D. Paraelectric HS phase: *ρ*_0_ = 0, *ρ*_1_ = *ρ*_2_ = *ρ*_3_ = 1/3 but **P** = 0. As the magnetic field increases, magnetoelectric coupling increasingly confines the electric dipoles to an axis that is different for each sublattice.

The calculated phase diagram in Fig. 5d reasonably matches the experimental data. In contrast to previous literature^34,40^, it includes magnetic anisotropy and the contribution to the free energy from electric dipoles. It also provides a mechanism for magnetoelectric coupling that emerges when the choice of the JT axis determines both the electric dipole direction and the magnetic easy axis. The curved phase boundary in the *H* − *T* space between phase C and phase D is explained by this magnetoelectric coupling. In contrast to ref. ^40^, our theory predicts an antipolar phase B and a polar phase C in agreement with our experiments.

In conclusion, we have explored how magnetoelectric coupling and multiferroic-like behavior can occur at a SST. We identified an electrically polar, and potentially ferroelectric, state in Mn(taa) where the coupling between different spin states on individual magnetic ions is primarily due to lattice strain from the different structural configuration of the molecules in the low and high spin states. This mechanism for coupling between magnetic behavior and electric polarization is different than most of the previously explored magnetoelectrics that rely on magnetic exchange coupling to create patterns in the spin directions such as ferromagnetism and antiferromagnetism. The SST creates mobile electric dipoles in the HS state that freeze in to trigger a structural phase transition to a polar state. This rich interplay of magnetic spin state ordering, elastic ordering of JT distortions, structural transitions and electric polarization in Mn(taa) can be controlled with temperature and magnetic field and demonstrates a promising route to magnetoelectric behavior. This demonstration of creating magnetoelectric behavior by means of an SST shows an *H*-induced electric polarization within a factor of ten of the record for any compound^46^.

There are a very large number of materials showing SSTs/spin crossovers, likely in the tens to hundreds of thousands, and these are mostly materials with organic ligands. An increasing number of SST materials order above room temperature making them attractive for practical applications. These materials have functionalities that are different from those of inorganic oxides, such as extreme sensitivity to pressure, light, and gas adsorption. These could be combined with magnetism and ferroelectricity to create various types of multifunctionality. Thus the discovery of magnetoelectric behavior in Mn(taa) opens a broad class of materials and mechanisms for future studies.

## Methods

### Sample synthesis

Mn(taa) was synthesized by mixing Mn(acac) (Strem, acac = acetylacetonate) and H_3_taa^49^ in CH_2_Cl_2_ and allowing the mixture to stir overnight. After filtering through celite, the solution was dried in vacuum, dissolved in THF, and again filtered through celite. Crystals were grown using vapor diffusion of hexanes into the THF solution. Unit cell parameters determined from single crystal X-ray diffraction were consistent with previous reports^28,33^. The orange-brown crystals were mm-sized with clearly-defined facets and tetrahedral geometries and the faces were identified as (110).

### Magnetic field sweep rates

High magnetic field experiments were conducted at the NHMFL facilities in Tallahassee, FL and Los Alamos National Lab, NM. Pulsed magnetic fields up to 60T were applied on millisecond timescales using resistive magnets powered by a capacitor bank with sweep rate up to 6 kTs^−1^. Quasi-DC magnetic fields up to 35 or 45T were applied in resistive and resistive-superconducting hybrid magnets with ramp rates of 0.08–0.17 Ts^−1^. Quasi-DC fields up to 14T were applied by superconducting magnets with (0.1 Ts^−1^) ramp rates at Los Alamos National Lab and Harvey Mudd College.

### Heat capacity measurements

Heat capacity was measured in a Quantum Design Physical Properties Measurement System (PPMS) using both a relaxation method and a heat sweep method at the sharp first-order jump at the SST for a collection of hundreds of single crystals, where each crystal has a [110] face in contact with the sapphire measurement plate via apeizon N grease.

### Magnetization measurements

The magnetization in 9 and 14T superconducting (quasi-DC) magnets was measured using vibrating sample magnetometers in PPMS systems on collections of single crystals. In pulsed fields, magnetization was measured on collections of single crystals in a plastic capsule secured with apeizon N grease. The magnetization was determined by a well-established technique of integrating the voltage in a dynamically compensated pickup coil, with in-situ sample-in/sample out background subtraction^43^. Magnetic field in pulsed magnets was measured by integrating the induced voltage in a copper coil and was calibrated using the de Haas-van Alphen oscillations of copper. Helium-3 and/or Helium-4 were used to thermalize the samples and a cernox thermometer recorded *T* before each field pulse.

### Electric polarization measurements

Δ*P* (*H*) in pulsed fields was measured on one single crystal at a time with silver epoxy contacts on parallel faces, and due to the delicacy of the crystals, the as-grown [110] faces were used. Δ *P*(*H*) was measured by an established technique^44,48,50–55^ in which backgrounds and pulsed-field artifacts have been shown to be negligible compared to the signal size. The change in surface charge was measured with a Stanford Research Systems 570 current-to-voltage converter and then integrated in time to extract Δ*P*(*H*). The electric polarization measurements in pulsed fields are more sensitive than in quasi-DC fields due to the large d*H*/d*t*. In the 45T DC hybrid magnet, Δ*P* (*H*) was measured by recording the current resulting from a change in surface charge on the contacts with a Keithley 6517A electrometer on one single crystal at a time. No voltage was applied in the data shown in the paper, however in other measurements we applied voltages up to 100 V either while cooling, or during the measurements to demonstrate no voltage dependence beyond a 1% effect consistent with the capacitance.

In the Supplementary Note 1 we discuss how we rule out artifacts in the surface charge measurements from conductivity, magnetostrictive, and triboelectric effects. Most of these are ruled out by the lack of a significant voltage dependence of the electric polarization and by the 10^−5^ loss tangent (greater than tera-ohm resistance of the mm-sized samples). In addition, we see similar electric polarization in fast and slowly-varying *H*, as well as in fixed *H* as a function of *T*, and the electric polarization change in DC measurements is consistent for up and down sweeps of *H*.

The dielectric constant in superconducting and quasi-DC magnets was determined by measuring the capacitance across silver epoxy contacts applied on opposing faces along the [110] axis of one single crystal at a time with an Anders-Hagerling 2700A capacitance bridge at 5 kHz and 30 V.

The temperature was recorded with Cernox thermometers mounted on a thin sapphire plate along with the sample. Measurements were conducted in Helium-4 or Helium-3 gas. The magnetic-field dependencies of the thermometers were corrected for and verified to be <1% in the region of where data was taken for DC fields, and measured at the beginning of the pulse for pulsed measurements.

Temperature changes in pulsed fields due to eddy currents are known to be negligible in our measurement setup, and magnetocaloric effects are calculated to be negligible, discussed in detail in the Supplementary Note 1.

## Supplementary information

Supplementary Information

## Data Availability

The data that support the findings of this study are available from the corresponding author upon reasonable request.
