# Supplementary material for: Magnetoelectric behavior via a spin state transition
Source: Nat Commun. 2019 Sep 6;10:4043. doi: 10.1038/s41467-019-11967-3 (PMC6731214; doi:10.1038/s41467-019-11967-3)
Supplement: Supplementary file 1 — Supplementary Information [file 41467_2019_11967_MOESM1_ESM.pdf]

Magnetoelectric behavior via a spin state transition

Chikara et. al.

Supplementary Information

Supplementary Notes

Supplementary Note 1. Artifacts to be considered in electric polarization measurements. We took care to rule out contribution to the electric polarization in Mn(taa) from the artifacts as described below.

Conductivity artifacts. In the ten year history of conducting pulsed-field electric polarization measurements in our group we have not seen pulsed field-induced artifacts nor backgrounds that are significant compared to this data except for an initial noise spike at 0 T due to the capacitor bank discharges and are removed from our measurement. We measured the loss tangent of the dielectric constant to be $< 10^{-5}$ below 50 K which limits conductivity artifacts. The mm-sized pyramidal samples had greater than tera-ohm resistances below 50 K. The measurements shown in the paper were conducted for zero applied voltage. We find that voltages up to 100 V applied during the electric polarization measurements create $\leq 1\%$ change in the measured electric polarization, which is a change consistent with the dielectric constant. Based on this result we can rule out artifacts in the electric polarization due to electric currents passing through the sample or due to magnetostriction, since such artifacts would scale linearly with applied voltage. We did not see significant changes in the electric polarization when cooling through the transition in an applied voltage of 100 V. Finally, we rule out spontaneous charge release or triboelectric effects[1] because the electric polarization change is reproducible for a given sample. Most importantly, the magnitude of the electric polarization change is reversible between up and down sweeps of temperature or magnetic field.

Magnetocaloric artifacts. In pulsed fields, the SST is pushed to higher fields than for quasi-DC fields due to the first-order nature of the phase transition and shows very significant hysteresis between the up and down sweeps. While magnetocaloric effects could account for part of this shift, the main shift between DC and pulsed phase diagrams occurs along the field axis, not the temperature axis, with critical fields achieved in pulsed fields that are not seen in DC fields for any temperature.

Supplementary Note 2. This section describes our theoretical model in detail. Here we provide a phenomenological 4-state Potts model to describe the spin state transition (SST). The four states are ρ_i, $i = 0, 1, 2, 3$ subject to the constraint $\sum_{i=0}^{3} \rho_i = 1$. The index $i = 0$

indicates the $S = 1$ state, and $i = 1, 2, 3$ are the three different ferrodistortive $S = 2$ states that have electric dipoles pointing in three directions as explained in the following. All the molecules can be grouped into four sublattices based on the direction of their major C_3 axes. In the HS state a JT elongation occurs that is mostly along the C_3 axis, but also has a small perpendicular component. This perpendicular component can point in one of three directions for any given sublattice, and it creates an electric dipole perpendicular to the original C_3 axis.[2,3] Each molecule in a given sublattice thus has a choice of three different directions for the electric polarization that lie transverse to the molecule's original undistorted C_3 axis of the low spin state. $\mathbf{n}_{ij}$ denotes the direction of the ith polarization axis in the jth sublattice, and $\sum_j \mathbf{n}_{ij} = 0$ by symmetry, for $i = 1, 2, 3$ and $j = 1, 2, 3, 4$. Each $\mathbf{n}_{ij}$ has a corresponding sublattice-resolved population ρ_{ij} ($\sum_{j=1}^{4} \rho_{ij} = \rho_i$) whose role will become clear below.

We write the total free energy density as a sum of three parts,

$$\mathcal{F} = \mathcal{F}_1 + \mathcal{F}_2 + \mathcal{F}_3. \qquad \text{(Supplementary Eq. 1)}$$

The first part, $\mathcal{F}_1$, is the same that has been published previously[2,3], and contains a Landau-type expansion in terms of the order parameters ρ_i, $i = 0, 1, 2, 3$, up to the second order, as well as the entropy of mixing,

$$\mathcal{F}_1 = J_0 \sum_{i=1}^{3} \rho_i^2 + 2J_1\rho_0 \sum_{i=1}^{3} \rho_i + \Delta \sum_{i=1}^{3} \rho_i + T \sum_{i=0}^{3} \rho_i \ln \rho_i. \qquad \text{(Supplementary Eq. 2)}$$

The first term in $\mathcal{F}_1$ is the ferrodistortive interaction between the high spin states, and $J_0 < 0$ is the ferrodistortive interaction constant. The second term describes the exclusion between the low and the high spin state, $J_1 > 0$ is the Ising-type demixing interaction between high spins and low spin states. Δ is the gap between the low spin and the high spin states.

The second part, $\mathcal{F}_2$, is the free energy density for the free spins in a magnetic field $\mathbf{H}$ via the Hamiltonian $\mathcal{H}_{ij}$ of a single molecule, where the subscript i denotes the spin state. If there is no inter-molecular interaction, this term is,

$$\mathcal{F}_2 = -T\rho_0 \ln\left[\mathrm{Tr}\left(e^{-\beta\mathcal{H}_0}\right)\right] - T \sum_{i=1}^{3}\sum_{j=1}^{4} \rho_{ij} \ln\left[\mathrm{Tr}\left(e^{-\beta\mathcal{H}_{ij}}\right)\right]. \qquad \text{(Supplementary Eq. 3)}$$

where the Hamiltonian $\mathcal{H}_{ij}$ are defined in terms of the applied magnetic field $\mathbf{H}$,

$$\mathcal{H}_{ij} = \begin{cases} - g_L \mu_B \mathbf{H} \cdot \mathbf{S}^{(\mathrm{L})} = \mathcal{H}_0^{(0)}, & i = 0 \\ - g_H \mu_B \mathbf{H} \cdot \mathbf{S}^{(\mathrm{H})} - D_{ij} (\mathbf{m}_{ij} \cdot \mathbf{S}^{(\mathrm{H})})^2 = \mathcal{H}_i^{(0)} - D_{ij} (\mathbf{m}_{ij} \cdot \mathbf{S}^{(\mathrm{H})})^2, & i = 1, 2, 3 \end{cases}$$

(Supplementary Eq. 4)

where g_L and g_H are the g-factor for the low and high spin state, respectively, μ_B is the Bohr magneton and $\mathbf{S}^{(\mathrm{L})}$ ($\mathbf{S}^{(\mathrm{H})}$) denotes spin operator for low (high) spin. $D_{ij}(\mathbf{m}_{ij} \cdot \mathbf{S}^{(\mathrm{H})})^2$ are the magnetic anisotropy energies. ρ_{ij} are sublattice occupation numbers, $\sum_{j=1}^{4} \rho_{ij} = \rho_i$. We are interested in the correlated deviations from $\rho_{ij} = \rho_i/4$ that will lead to a net dipole moment $\mathbf{P}$. Such cases can be approximated by a linear dependence on the polarization,

$$\rho_{ij} = \rho_i \left[\frac{1}{4} + \eta (\mathbf{n}_{ij} \cdot \mathbf{P}) \right], \qquad \text{(Supplementary Eq. 5)}$$

The polarization $\mathbf{P}$ is given by

$$\sum_{i=1}^{3} \sum_{j=1}^{4} \rho_{ij} P_{\mathrm{tr}} \mathbf{n}_{ij} = \mathbf{P}, \qquad \text{(Supplementary Eq. 6)}$$

where P_{tr} is the magnitude of the transverse polarization of a single molecule in the high spin state.

$\mathrm{Tr}\left(e^{-\beta H_{ij}}\right)$ has a simple form for $S = 2$ as follows,

$$\begin{aligned} \mathcal{F}_2 + \mathcal{F}_3 = & - T \sum_{i=0}^{3} \rho_i \ln \left[\mathrm{Tr}\left(e^{-\beta H_i^{(0)}} \right) \right] - \frac{J_2}{4} \left[2 + \frac{7}{5} (\beta \mu_B g_H H)^2 \right] \sum_{i=1}^{3} \rho_i^2 \\ & + a \left\{ \left[T - c - \frac{\lambda}{a} \left[2 + \frac{7}{5} (\beta \mu_B g_H H)^2 \right] \sum_{i=1}^{3} \rho_i^2 \right] P^2 + b P^4 \right\}, \end{aligned}$$

(Supplementary Eq. 7)

where H is the magnitude of the magnetic field, θ_{ij} is the angle between the magnetic field and anisotropy axis, and we assume that the anisotropy energy is small compared to the temperature. Keeping the lowest order field terms in the anisotropy energy, we find,

$$\ln \left[\mathrm{Tr}\left(e^{-\beta \mathcal{H}_{ij}} \right) \right] = \ln \left[\mathrm{Tr}\left(e^{-\beta \mathcal{H}_i^{(0)}} \right) \right] + \left[2 + \frac{7}{5} (\beta \mu_B g_H H)^2 \right] \beta D_{ij} \cos^2 \theta_{ij}.$$

(Supplementary Eq. 8)

$\mathcal{F}_2$ becomes,

$$\mathcal{F}_2 = -T \sum_{i=0}^{3} \rho_i \ln \left[\mathrm{Tr}\left(e^{-\beta \mathcal{H}_i^{(0)}} \right) \right] - \left[2 + \frac{7}{5} (\beta \mu_B g_H H)^2 \right] \sum_{i=1}^{3} \sum_{j=1}^{4} \rho_{ij} D_{ij} \cos^2 \theta_{ij}.$$

(Supplementary Eq. 9)

The magnitude and axis of the anisotropy for each molecule is dynamically dependent on its environment, which is correlated to ρ_{ij}. The simplest dependence is a linear relationship, $D_{ij}\cos^2\theta_{ij} \approx D + J_2\rho_{ij}$. We obtain,

$$\begin{aligned}\sum_{i=1}^{3}\sum_{j=1}^{4}\rho_{ij}\left(D + J_2\rho_{ij}\right) &= J_2\sum_{i=1}^{3}\sum_{j=1}^{4}\rho_i^2\left[\frac{1}{16} + \frac{\eta}{2}\mathbf{n}_{ij}\cdot\mathbf{P} + \eta^2(\mathbf{n}_{ij}\cdot\mathbf{P})^2\right] + D \\ &= \frac{J_2}{4}\sum_{i=1}^{3}\rho_i^2 + J_2\eta^2\sum_{i=1}^{3}\rho_i^2\sum_{j=1}^{4}(\mathbf{n}_{ij}\cdot\mathbf{P})^2 + D \\ &= \frac{J_2}{4}\sum_{i=1}^{3}\rho_i^2 + \lambda\sum_{i=1}^{3}\rho_i^2 P^2 + D \qquad \text{(Supplementary Eq. 10)}\end{aligned}$$

where $\lambda = J_2\eta^2\sum_{j=1}^{4}\left(\mathbf{n}_{ij}\cdot\hat{\mathbf{P}}\right)^2$ is assumed to be independent of i and $\hat{\mathbf{P}}$. The last term does not depend on the order parameters so it can be dropped from the free energy.

The third part, $\mathcal{F}_3$, contains the electric polarization,

$$\mathcal{F}_3 = a[(T-c)P^2 + bP^4]. \qquad \text{(Supplementary Eq. 11)}$$

It describes ferroelectric-paraelectric phase transition.

The final form for $\mathcal{F}_2 + \mathcal{F}_3$ is,

$$\begin{aligned}\mathcal{F}_2 + \mathcal{F}_3 = &-T\sum_{i=0}^{3}\rho_i\ln\left[\mathrm{Tr}\left(e^{-\beta H_i^{(0)}}\right)\right] - \frac{J_2}{4}\left[2 + \frac{7}{5}(\beta\mu_B g_H H)^2\right]\sum_{i=1}^{3}\rho_i^2 \\ &+ a\left\{\left[T - c - \frac{\lambda}{a}\left[2 + \frac{7}{5}(\beta\mu_B g_H H)^2\right]\sum_{i=1}^{3}\rho_i^2\right]P^2 + bP^4\right\},\end{aligned}$$

(Supplementary Eq. 12)

The term $\rho_i^2 H^2 P^2$ comes from the magnetic anisotropy, and gives the field-dependence of electric polarization inside the HS phase.

Supplementary Discussion

The comparison between the theoretical and experimental phase diagrams is shown in Fig. 5(d) of the main text. The phase diagram is not changed by parameter a between 0 and 0.1 $\mathrm{m}^4/\mathrm{mC}^2$, and additional experimental data is needed to determine it. Other parameters fitted to the phase diagram are ($k_B = 1$): $J_0 = -41$ K, $J_1 = 125$ K, $J_2 = -0.3$ K, $\Delta = 90$ K, $c = 47$ K, $\lambda/a = -6.2$ K, $b = 60$ K·$\mathrm{m}^4/\mathrm{mC}^2$.

The four phases in Fig. 5(d) of the main text are identified as:

A. Low spin phase: $\rho_0 = 1$, $\rho_1 = \rho_2 = \rho_3 = 0$ (lower left).

B. HS phase with net $P = 0$: $\rho_0 = 0$ and one of $\rho_{1,2,3} \approx 1$ (upper left). Without loss of generality we assume $\rho_1 \approx 1$. $\rho_{1j} \approx 1/4$ and because $\sum_j \mathbf{n}_{ij} = \mathbf{0}$, we have $\mathbf{P} = \mathbf{0}$, i.e., because each molecule has a different orientation and is distorted along their own JT axis, their dipoles cancel each other by symmetry, resulting in a nearly zero total polarization.

C. Polar high spin phase: $\rho_0 = 0$, $\rho_1 = \rho_2 = \rho_3 = 1/3$ with $P \neq 0$ (upper middle). At higher temperatures, a polar high spin phase appears. In this phase, $\rho_{1,2,3}$ are distributed evenly to maximize the entropy. The electric dipole moment is formed due to ordering of the electrically polar distortions between the four sublattices. The phase boundary between B and C has a negative slope, which is the reason for the small negative value of J_2.

D. Paraelectric high spin phase with residual electric polarization: $\rho_0 = 0$, $\rho_1 = \rho_2 = \rho_3 = 1/3$ but $P = 0$ (upper right). As the temperature is increased, a ferroelectric-paraelectric phase transition occurs. Even though both phases C and D have $\rho_{1,2,3} = 1/3$, the ρ_{ij}'s are different. In the polar phase, the ρ_{ij}'s are not uniform for different sublattices, and this deviation from $\rho_i/4$ gives the polarization $\mathbf{P}$, while in the high-temperature paraelectric phase, ρ_{ij} are uniform. Besides SST-induced polarization, the strongly field-dependent ferroelectric-paraelectric transition temperature is also a manifestation of magnetoelectric coupling.

Supplementary References

[1] Wang, Z. L. Triboelectric nanogenerators as new energy technology for self-powered systems and as active mechanical and chemical sensors. *ACS Nano* **7**, 9533-9557 (2013)

[2] Nakano, M., Matsubayashi, G. -e. & Matsuo, T. Dynamic Jahn-Teller character of Manganese(III) spin-crossover complex [Mn(taa)] (H_3taa = tris$(1-(2-$azolyl$)-2-$azabuten$-4-$yl)amine). *Adv. Quantum Chem.* **44**, 617 (2003)

[3] Nakano, M., Matsubayashi, G. -e. & Matsuo, T. Dielectric behavior of manganese(III) spin-crossover complex [Mn(taa)] *Phys. Rev. B* **66**, 212412 (2002)

Supplementary Figures

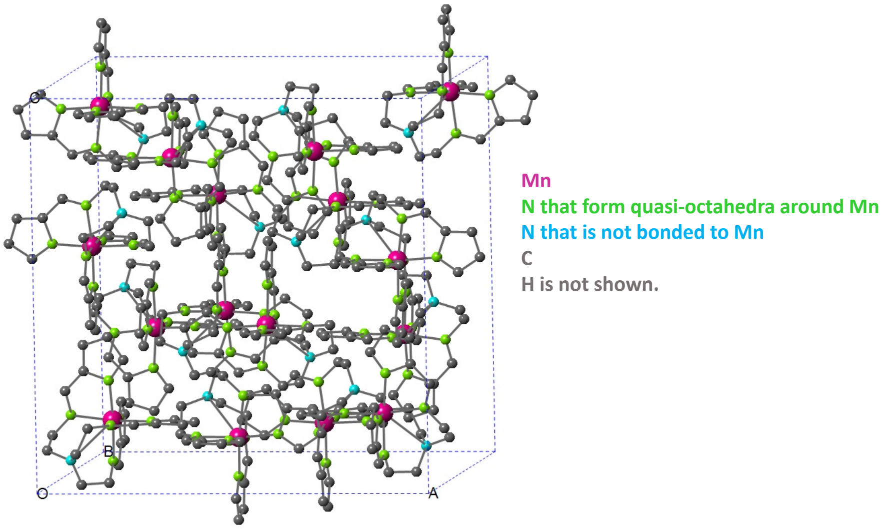

Supplementary Figure 1. A unit cell of Mn(taa). The Mn ions are quasi-octahedrally coordinated to six green N ions. These six N ions occupy two different crystallographic sites (three each). The blue N is non-coordinated to the Mn ion and breaks the rotational symmetry of the Mn(taa) molecule.

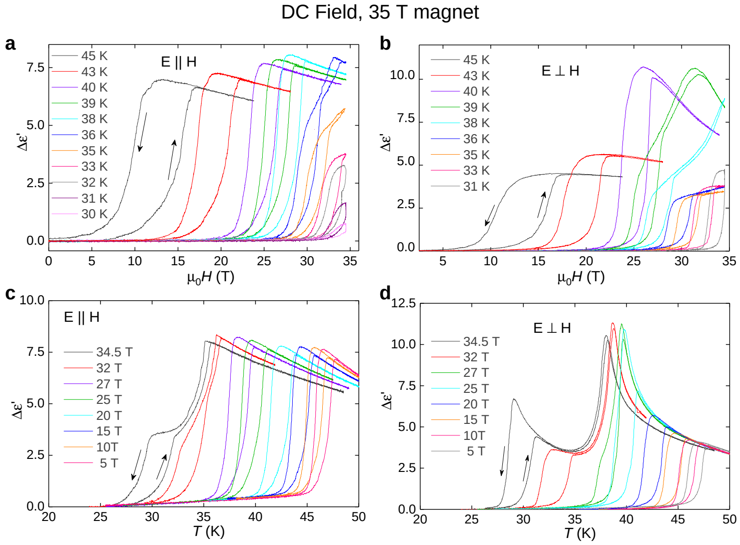

Supplementary Figure. 2. The dielectric constant at 35 T. The dielectric constant $\epsilon'(H,T)$ for the electric field $\mathbf{E} \parallel \mathbf{H}$ and $\mathbf{E} \perp \mathbf{H}$ configurations in 35 T quasi-DC field is qualitatively consistent with the 45 T data on a different sample shown in Supplementary Figure 3.

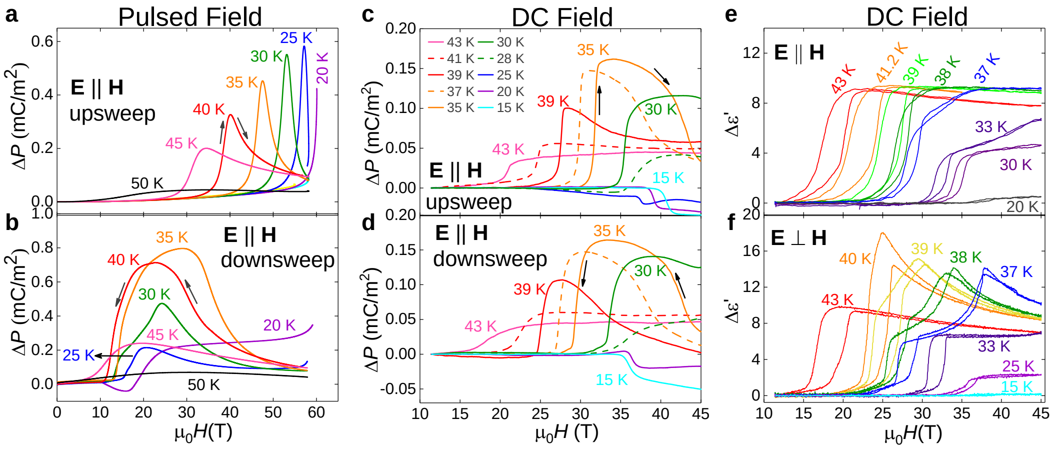

Supplementary Figure 3. Magnetic field induced electric polarization measured in pulsed and quasi-DC magnetic fields. (a) and b) Electric polarization $\Delta P(H)$ for different T during the upsweep and downsweep of the magnetic field H integrated from measured dP/dt data. (c) and (d), $\Delta P(H)$ measured in quasi-DC fields up to 45 T at different T for the upsweep and downsweep. (e) and (f), Dielectric constant $\Delta\epsilon'(H)$ measured in quasi-DC field up to 45 T for $\mathbf{E} \parallel \mathbf{H}$ and $\mathbf{E} \perp \mathbf{H}$ respectively, where $\mathbf{E}$ is the electric polarization/electric field measurement axis. $\Delta P(H)$ as a function of T in quasi-DC magnetic field are consistent and shown in Supplementary Figure 4. In all measurements, $\mathbf{P} \parallel$ **[110]** of the cubic crystal and relative orientation of $\mathbf{H}$ and $\mathbf{E}$ (where $\mathbf{E} \parallel \mathbf{P}$) are indicated.

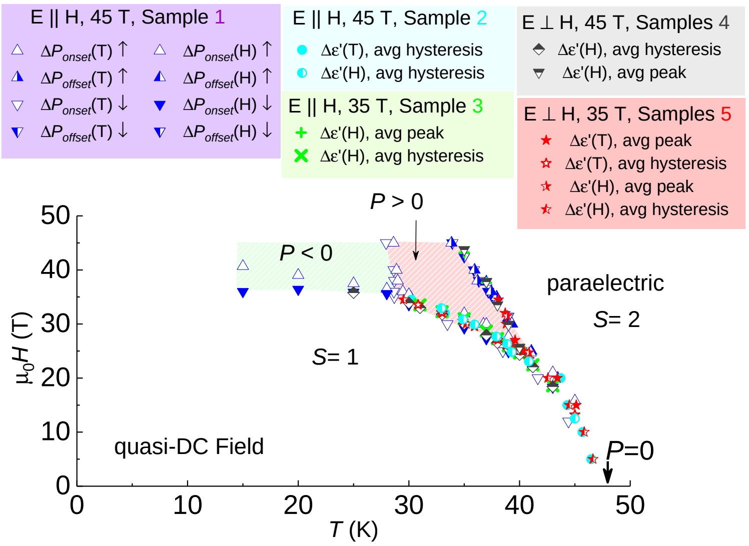

Supplementary Figure 4. The quasi-DC magnetic field phase diagram. It includes data points derived from $\Delta P(H,T)$ and $\epsilon'(H,T)$ where $\mathbf{E}$ is the electric polarization or dielectric constant measurement axis. We show $\mathbf{E} \parallel \mathbf{H}$ and $\mathbf{E} \perp \mathbf{H}$ for different samples measured in 35 T and 45 T quasi DC magnetic fields. The red and green shaded regions correspond to the red and green regions in Fig. 5 where the spontaneous electric polarization has opposite signs. Average peak (hysteresis) refers to the average of the peak position (transition field) for the up and down sweep of magnetic field.

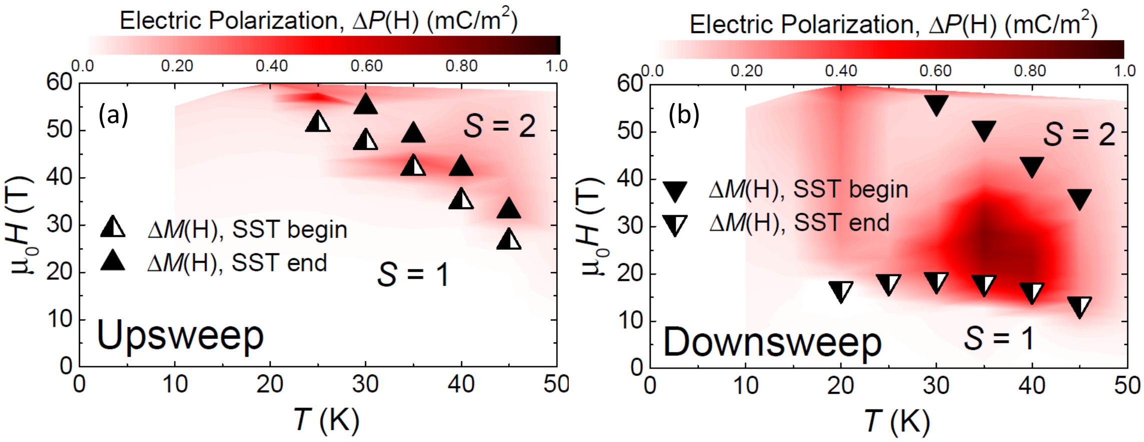

Supplementary Figure 5. Magnetic field H vs temperature T phase diagram of Mn(taa) for $\mathbf{E} \parallel \mathbf{H}$ in pulsed magnetic fields for up and downsweeps. The color-mapped regions show the spontaneous electric polarization. The black data points in left and right panels correspond to the beginning and end of the SST in the pulsed-field magnetization.

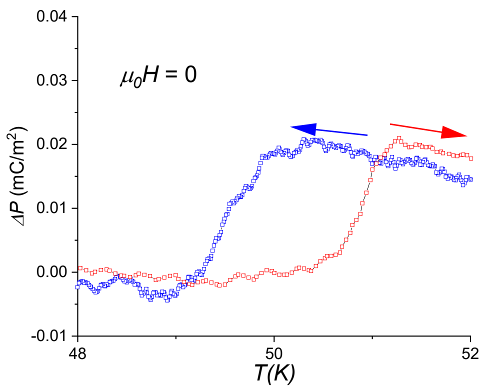

Supplementary Figure 6. Electric Polarization ΔP vs temperature T for Mn(taa) at zero magnetic field. At $H = 0$, the electric polarization change at the temperature induced SST is roughly 0.02 mC/m^2.
